# **A community-acquired Legionnaires’ disease outbreak caused by *Legionella pneumophila* serogroup 2**: **an uncommon event, Italy, August to October 2018**

**DOI:** 10.2807/1560-7917.ES.2021.26.25.2001961

**Published:** 2021-06-24

**Authors:** Maria Scaturro, Maria Cristina Rota, Maria Grazia Caporali, Antonietta Girolamo, Michele Magoni, Daria Barberis, Chiara Romano, Danilo Cereda, Maria Gramegna, Antonio Piro, Silvia Corbellini, Cinzia Giagulli, Giovanni Rezza, Arnaldo Caruso, Maria Luisa Ricci

**Affiliations:** 1 Department Infectious Diseases, Istituto Superiore di Sanità, Rome, Italy; 2 Agency for Health Protection of Metropolitan Area of Milan, Milan, Italy; 3 Public Health Laboratory, Agency for Health Protection of Brescia, Brescia, Italy; 4 Public Health Managment of Welfare, Lombardy Region, Milan, Italy; 5 Agency for Health Protection of Valpadana, Mantova, Italy; 6 Microbiology and Virology unit, Spedali Civili Brescia Hospital, Brescia, Italy; 7 Department of Molecular and Translational Medicine, Microbiology unit, University of Brescia, Brescia, Italy; 8 Department of Health Prevention, Ministery of Health, Ministry of Health, Rome, Italy; 9 Department of Experimental and Applied Medicine, Section of Microbiology, Spedali Civili Hospital, Brescia, Italy

**Keywords:** *Legionella*, Legionnaires' disease, outbreak, river, Italy

## Abstract

In September 2018 in Brescia province, northern Italy, an outbreak of Legionnaires' disease (LD) caused by *Legionella pneumophila* serogroup 2 (*Lp2*) occurred. The 33 cases (two fatal) resided in seven municipalities along the Chiese river. All cases were negative by urinary antigen test (UAT) and most were diagnosed by real-time PCR and serology. In only three cases, respiratory sample cultures were positive, and *Lp2* was identified and typed as sequence type (ST)1455. In another three cases, nested sequence-based typing was directly applied to respiratory samples, which provided allelic profiles highly similar to ST1455. An environmental investigation was undertaken immediately and water samples were collected from private homes, municipal water systems, cooling towers and the river. Overall, 533 environmental water samples were analysed and 34 were positive for *Lp*. Of these, only three samples, all collected from the Chiese river, were *Lp2* ST1455. If and how the river water could have been aerosolised causing the LD cases remains unexplained. This outbreak, the first to our knowledge caused by *Lp2*, highlights the limits of UAT for LD diagnosis, underlining the importance of adopting multiple tests to ensure that serogroups other than serogroup 1, as well as other *Legionella* species, are identified.

## Background

The first large outbreak of Legionnaires’ disease (LD) was identified in Philadelphia in 1976. Since this event, a large number of outbreaks and sporadic cases have been reported, mainly caused by *Legionella pneumophila* serogroup 1 (*Lp1*) [1-4]. Indeed, *Lp1* is the main cause of LD worldwide and is responsible for over 80% and 70% of laboratory-confirmed legionellosis cases in Europe and United States, respectively, although other species may also infect humans [5,6]. For example, in New Zealand and Scotland, *Legionella longbeachae* represents a key threat to human health, causing 30–55% of LD cases [5]. In 2019, in Europe, incidence of LD cases was 2.2 per 100,000 inhabitants, showing an increasing trend compared with previous years [6]. According to the European Centre for Disease Prevention and Control (ECDC) Surveillance Atlas of Infectious Disease, Italy was the country in the European Union (EU) in 2019 that reported the highest number of LD cases (3,143) and was second in incidence, after Slovenia [7].

Outbreaks of non-*Lp1* serogroups have been rarely documented. These serogroups are widespread in all man-made water systems; they mainly cause sporadic cases and are often responsible for hospital-associated cases [8-13]. Since the beginning of epidemiological surveillance in Italy in 1983, few sporadic LD cases caused by non-*Lp1* serogroups have been reported [14]. According to the EU case definition, laboratory criteria for LD case confirmation must include at least one of the following three: (i) isolation of *Legionella* spp. from respiratory secretions or any normally sterile site, (ii) detection of *Lp* antigen in urine, (iii) a significant rise in specific antibody level to *Lp1* in paired serum samples [15]. Currently, LD diagnosis is primarily based on the detection of urinary antigen and most of the commercially available tests are specifically designed to diagnose infections caused by *Lp1.* Thus, they are poorly sensitive in detecting infections caused by non-*Lp1* strains [16]. For this reason, cases of LD caused by non-*Lp1* serogroups or by *L.* non-*pneumophila* often remain undetected [17]. Although the detection of *Legionella* spp. nucleic acid in respiratory secretions, lung tissue or any normally sterile site defines a probable case according to the EU case definition, PCR has been successfully introduced in several countries as a routine diagnostic method and the number of reported LD cases has markedly increased [18-20]. This is consistent with the high sensitivity and specificity of PCR to detect all *Lp* serogroups and *L.* non-*pneumophila* species in respiratory samples [21,22]. As the majority of *Lp* infections are caused by *Lp1* strains, a real-time PCR assay has been validated by the European Society of Clinical Microbiology and Infectious Diseases Study Group for Legionella Infections with the aim of accurate detection in clinical specimens [21]. The assay is designed to simultaneously detect *Lp* serogoups 1–15 by targeting the *mip* gene, and the *Lp1* serogroup specifically by targeting the *wzm* gene. Thus, this multiplexed assay allows us to recognise all *Lp* serogroups and distinguish *Lp1*.

### Outbreak detection

In 2018, between the end of August and the first 2 weeks of October, a sudden increase of LD cases was reported in Brescia province, most notably in seven municipalities located along the Chiese river. Within the first few days of September, the Agency for Health Protection of the Brescia province was notified of 665 pneumonia cases requiring hospitalisation. Cases were immediately tested for *Legionella* by urinary antigen test (UAT), which gave negative results in more than 90% of cases. Only after real-time PCR was performed on respiratory secretions of UAT-negative cases was *L. pneumophila* detected. When respiratory secretions were available, culture examination was also requested. Immediately, a multidisciplinary team, which consisted of public health professionals, epidemiologists, medical doctors and microbiologists, was established to conduct epidemiological and environmental investigations to identify the source of the outbreak and to adopt the needed control measures.

The objective of our report is to describe epidemiological, environmental and microbiological investigations and report factors that contributed to the outbreak.

## Methods

### Epidemiological investigation

The first LD case was detected 30 August 2018. The epidemiological investigation started few days later, on the first day of September and lasted until 18 October 2018. Since *Legionella* was the pathogen most suspected to cause the outbreak, an outbreak case definition was formulated to distinguish LD from pneumonia of a different origin. A case of LD was included in the outbreak if they resided in Brescia province, presented with clinical symptoms of pneumonia and had laboratory confirmation for *Legionella* with at least one diagnostic test, according to the EU case definition [15].

Nosocomial and travel-related LD cases were excluded. Case finding included mandatory notifications and requests for information to local health authorities, general practitioners and hospitals present in the area. General practitioners and hospital physicians were informed of the ongoing outbreak and were asked to test all pneumonia cases for *Legionella* in order to enhance surveillance.

Cases were required to answer a standardised questionnaire addressing health conditions (presence of chronic diseases, smoking and alcohol habits, corticosteroids therapy and/or chemotherapy), places visited and routes travelled within their town vicinity, as well as usual social activities. The cases were mapped using a geographic information system to detect spatial movement patterns .

### Microbiological analysis of clinical samples

Following the alert that the increase in pneumonia cases was attributable to *Legionella* infection, medical staff of all hospitals in Brescia province were asked to collect urine, respiratory secretions and sera from all cases admitted with pneumonia in order to test for LD. UAT (Legionella K-SeT, Coris Bioconcept, Gembloux, Belgium), culture of respiratory secretions or pulmonary tissues, real-time PCR and serology were used to diagnose cases.

Culture examination was carried out using agar plates with buffered charcoal yeast extract (BCYE-α; Thermo Fisher Scientific, Altrincham, United Kingdom (UK)) and selective antibiotic medium Modified Wadowsky–Yee (MWY) with BCYE growth supplement (Thermo Fisher).

As a first screen, respiratory secretions were analysed using the FTD atypical CAP kit (Fast Track Diagnostics, Luxembourg), a multiplex real-time PCR for detection of *L. pneumophila*/*L. longbeachae*, *Mycoplasma pneumoniae*, and *Chlamydophila pneumoniae*. Subsequently, real-time PCR on respiratory secretions and lung biopsy fragments was also performed using primers and probes for the *wzm* gene specific for *Lp1*, and the *mip* gene, which recognises all *Lp* serogroups, as previously described [21]. In addition, nested sequence-based typing (SBT) was directly applied to real-time PCR positive respiratory samples, according to the protocol previously described [22]. Serology was performed using an indirect immunofluorescence assay commercial kit (Bios GmbH, Munich, Germany). A positive single high titre serum was determined by microscopy when fluorescence (level of 1+ to 3+) was observed compared with a negative control; seroconversion was established when an increased level of fluorescence (3+) was detected in the convalescent serum compared with the serum collected in the acute phase of the disease.

Although single high titre for non-*Lp1* serogroups is not a sufficient criterion for defining an LD case according to the EU case definition, based on epidemiological considerations, we decided to include five pneumonia cases in the outbreak diagnosed by a single high titre.

### Microbiological analysis of environmental samples

To identify the source of infection, the local health personnel inspected a large number of possible sources located in the Brescia province, particularly in the municipalities located along the Chiese river. Water samples (1L) were collected from drinking water taps of cases’ homes, shopping centres, healthcare facilities, business and public buildings, from basin and circulating water of cooling towers of industrial sites and from wells and pipes of the municipal water network. The Chiese river and its irrigation canals that were adjacent to the municipalities where the most of cases occurred were also sampled. Water sample culturing was performed according to International Organization for Standardization (ISO) 11731:2017, using a detection limit of 100 colony-forming units ((CFU)/L) [23]. *Legionella* colonies isolated from both clinical and environmental samples were identified by latex agglutination test (Oxoid, Thermo Fisher Scientific). Serogroups of *L. pneumophila* were determined using monoclonal antibodies specific for each *Lp* serogroup (provided by the Medical Faculty, Institute of Medical Microbiology and Hygiene, Technical University Dresden, Dresden, Germany). Genotyping by SBT was used to identify the sequence type (ST) [24] and *Lp1* colonies were sub-grouped by monoclonal antibodies, according to the Dresden panel [25].

### Ethical statement

Ethical approval for the study was not necessary because the data used were collected as part of the infectious disease surveillance programmes defined by national legislation.

## Results

### Epidemiological investigation

Between 30 August and 18 October 2018, we detected 88 LD cases residing in the Brescia local health unit territory who were identified with at least one positive diagnostic test (UAT, culture, real-time PCR, or serology (seroconversion or single high titre)). Epidemiological investigation showed that the median age of cases was 60.6 years (range: 19–91), the ratio of men/women was 2.4:1 (62/26), and 35% of cases (31/88) had risks factors, such as diabetes, cardiopathy, or chronic, or autoimmune or immunosuppressive diseases. Comparing LD incidence in 2018, disaggregated by municipality of residence within the province, with the same period in 2015–17, a significant increase in the incidence rate was observed in seven municipalities located along the Chiese river, an area with a total population of 57,000 inhabitants (Table 1).

**Table 1:** Cases and incidence rate of Legionnaires’ disease cases per million inhabitants, Brescia province, Italy, 2015–2018

Area	Population	2015		2016		2017		2018	
		Cases	Incidence rate^a^ (95% CI)	Cases	Incidence rate^a^ (95% CI)	Cases	Incidence rate^a^ (95% CI)	Cases	Incidence rate^a^ (95% CI)
**Brescia province**	1,177,365	39	33.1 (23.6–45.3)	50	42.5 (31.5–56.0)	67	56.9 (44.1–72.3)	143	121.5 (102.4–143.1)
**Seven municipalities** **along Chiese river**	57,009	0	0 (0–64.7)	3	52.6 (19.9–153.8)	2	35.1 (4.2–126.7)	39	684.1 (486.5–935.1)
**Remaining** **municipalities**	1,120,356	39	34.8 (24.8–47.6)	47	42.0 (30.8–55.8)	65	58.0 (44.8–63.9)	104.0	92.8 (75.8–112.5)

CI: confidence interval.

^a^ Incidence rates are presented per 1,000,000 inhabitants.

In these seven municipalities in 2018, LD incidence rate had increased nearly 20 times compared with the previous year (684.1/1,000,000 vs 35.1/1,000,000 inhabitants). In the remaining municipalities, incidence only increased by 1.5 times (92.8/1,000,000 in 2018 vs 58.0/1,000,000 in 2017). This comparison therefore confirmed that the suspected LD outbreak involved only these seven municipalities. 

During the study period, 33 cases were diagnosed among the residents living along the Chiese river. The remaining 55 cases detected in the study period lived in other municipalities in the Brescia province and represented the number of sporadic cases reported annually by this province, shown by an LD incidence rate of 121.5/1,000,000 in 2018 compared with 56.9/1,000,000 inhabitants in 2017 (Table 1). For this reason, they were not considered part of the outbreak.

**Figure 1:**
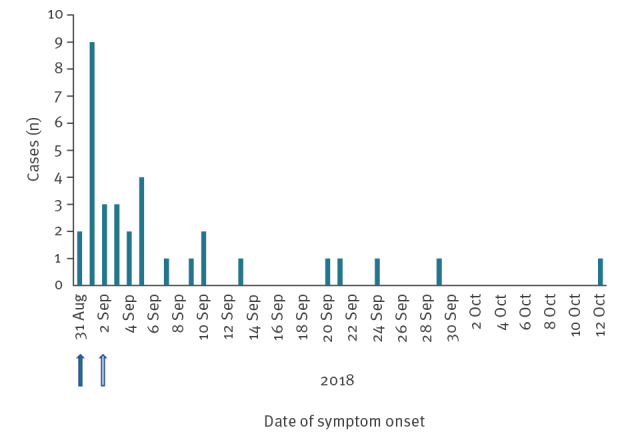
Epidemic curve of Legionnaires’ disease cases by date of symptom onset in residents of seven municipalities along the Chiese river, Brescia province, Italy, August–October 2018 (n = 33)

According to the epidemic curve, the outbreak occurred between the end of August and the first half of October 2018 (Figure 1). All 33 cases required hospitalisation and two cases died. 

Number of cases is reported on the date of symptoms onset. The solid blue arrow indicates the index case of the outbreak. The open blue arrow indicates the start of active case finding.

When we analysed the cases residing in the seven municipalities and those residing in the rest of the province separately, we found differences in the epidemiological characteristics (Table 2). The most remarkable difference was that the UAT was negative in all 33 cases residing in the municipalities located along the Chiese river.

**Table 2:** Comparison of outbreak vs non-outbreak cases of Legionnaires’ disease, Brescia province, Italy, August–October 2018 (n = 88)

Characteristics	Outbreak cases n = 33	Non-outbreak cases n = 55
Age in years (mean; range)	66.4 (43-89)	57.1 (26-93)
Men/women (ratio)	23/10 (2.3:1)	39/16 (2.4:1)
Laboratory diagnosis		
Urinary antigen test	0	25
Real-time PCR	24	30
Culture	3	0
Seroconversion	1	0
Single high titre	5	0
Underlying medical conditions	13	17

### Laboratory investigation of clinical and environmental samples

Diagnostic tests were performed on the 33 LD cases included in the outbreak. For 3 cases, *L. pneumophila* colonies were isolated by culture of respiratory samples, identified to be *L. pneumophila* serogroup 2 (*Lp2*); two were also positive by real-time PCR. In 24 cases, at least one of the two real-time PCR assays was positive; in 15 of these, the real-time PCR test distinguishing *Lp 1–15* serogroups from *Lp1* provided the presence of *Lp 2–15* DNA (Table 3). The two cases who died had respiratory secretions that were positive by real-time PCR, while lung biopsy fragments were negative for both real-time PCR and culture.

**Table 3:** Diagnosis and typing of clinical samples from Legionnaires’ disease cases residing along the Chiese river, Brescia province, Italy, August–October 2018 (n = 33)

Case	Municipality of residence along Chiese river	UAT	Culture	Real-time PCR^a^	Real-time PCR *Lp2–15* ^b^	SBT/Nested-SBT	Serology
1	A	N	N	P	P	No amp.	
2	A	N	N	P	ND	No amp.	
3	B	N	N	N	ND	No amp.	HT
4	B	N	N	P	ND	No amp.	
5	C	N	P	N	P	3,5,1,7,14,9,50 (1455)	
6	C	N	N	N	P	3,5,0,7,0,9,50 (1455?)	
7	C	N	N	P	P	No amp.	
8	C	N	N	P	P	No amp.	
9	C	N	N	N	ND	No amp.	SC
10	C	N	N	N	P	No amp.	
11	C	N	N	P	ND	No amp.	
12	C	N	N	P	ND	No amp.	
13	D	N	N	P	ND	No amp.	
14	E	N	N	P	P	No amp.	
15	E	N	N	P	P	3,5,1,7,0,9,50 (1455?)	
16	E	N	N	P	P	3,0,-1,0,14,1,50	
17	E	N	N	P	P	No amp.	
18	E	N	N	P	P	No amp.	
19	E	N	N	P	P	No amp.	
20	E	N	N	P	P	No amp.	
21	E	N	N	P	P	3,5,1,7,0,9,50 (1455?)	
22	E	N	N	N	ND	No amp.	HT
23	E	N	N	N	ND	No amp.	HT
24	E	N	N	N	ND	No amp.	HT
25	E	N	N	P	ND	No amp.	
26	E	N	N	P	ND	No amp.	
27	E	N	N	P	ND	No amp.	
28	F	N	P	P	P	3,5,1,7,14,9,50 (1455)	
29	F	N	N	N	ND	No amp.	HT
30	F	N	N	P	ND	No amp.	
31	F	N	N	P	ND	No amp.	
32	F	N	N	P	ND	No amp.	
33	G	N	P	N	ND	3,5,1,7,14,9,50 (1455)	

HT: single high titre; N: negative; ND: not determined; No amp.: no amplification: P: positive; SBT: sequence-based typing; SC: seroconversion; UAT: urinary antigen test.

^a^ Real-time PCR was performed using a commercial kit, as denoted in Methods.

^b^ Real-time PCR was performed according to Mentasti et al. [21].

Serology demonstrated seroconversion in one case and a single high titre in five cases, all for non-serogroup 1 *L. pneumophila*. Thus, according to the EU case definition, of the 33 LD cases, three were confirmed and 25 were probable cases. The remaining five cases, diagnosed by single high titre, were included in the outbreak based on epidemiological considerations. The *Lp2* clinical isolates were typed as ST1455. In three additional cases that were positive for *Lp2–15* by real-time PCR, nested-SBT provided allelic profiles highly similar to ST1455, lacking only one or two alleles. For Case 16, the allelic profile lacked three alleles, and had a difference in the *pro*A locus (i.e. for *pro*A 9 instead of 1) (Table 3).

From the beginning of September to the end of the outbreak (18 October 2018), 533 water samples were collected and analysed by culture; only 34 (6.4) were positive for *Lp* at a concentration ranging between 400 and 10,000 CFU/L (Table 4).

**Table 4:** *Legionella pneumophila* detection and typing from investigated sampling sites in seven municipalities along the Chiese river, Brescia province, Italy, August–October 2018 (n = 533)

Sampling site	Total samples (n = 533)	*Lp*-positive samples (n = 34)	CFU/L range	Serogroup	ST
Cases’ homes	262	12	400–6,500	1,3, 4, 5, 6, 8,10	1
Industrial wells	54	2	3,000–4,500	1	12
Industrial cooling towers	102	10	400–10,000	6,1	2737, 2740, 2741
Chiese river	14	7	2,000–8,000	2	1455, 80
Sporting and accommodation sites	18	3	400	8	ND
Municipal water and wells	83	0	0	0	ND

CFU: colony-forming unit; L: litre; ND: not determined; ST: sequence type.

At least five *L. pneumophila* colonies of each sample were tested for serogroup. Two colonies were typed for ST.

Ten cases’ homes, and seven samples collected along the Chiese river and its irrigation canals were found contaminated by *Lp* non-serogroup 1 (Table 4). A minimum of five *Lp* colonies per sampling site were typed to determine serogroup and two colonies to determine the ST (Table 4). Seven of 14 water samples collected from the Chiese river were positive for *Lp2*. For each of these positive samples, five colonies were tested for serogroup and two were typed by SBT. Among the colonies of *L. pneumophila* serogroup 2, ST1455 was detected in three different samples while the others were ST80. Three industrial cooling towers were contaminated with *Lp* serogroups 1 and 6 (up to 10,000 CFU/L), underscoring the risk of bacterium proliferation and transmission without proper treatment and maintenance.

## Outbreak control measures

Prevention and control actions were implemented following a notice of the increase and were aimed at significantly reducing the risk of contracting the disease. The following interventions were adopted: (i) citizens residing in the outbreak area were informed of the best practices for *Legionella* control at home and asked to apply them, (ii) disinfection of all industrial and non-industrial plants that might give rise to aerosols was requested, (iii) a correct use of the Chiese river water was recommended, taking into account periods of drought and guaranteeing the minimum vital flow for the ecological balance of the river itself, (iv) European Legionnaires’ disease Surveillance Network was promptly informed by email in order to identify LD cases among European citizens who had travelled in the epidemic area. From 18 October 2018, the incidence of LD cases returned to the level of the previous years, indicating that the epidemic was over and demonstrating the effectiveness of the control measures applied. One of the greatest points of criticism faced during the outbreak investigation was the detection of *Lp2* in cooling towers, which highlighted the lack of a cooling tower register. As a result, the local health authority decided to enact a regional law aimed at implementing a mandatory notification of cooling towers, as recommended by the national *Legionella* guidelines [26].

## Discussion

In Italy between August and October 2018, a LD outbreak consisting of 33 cases caused by *Lp2* occurred in seven municipalities of the Brescia province located along the Chiese river. Indeed, all 33 cases were negative by UAT, and in three cases, culture-confirmed *Lp2* ST1455 was isolated. To the best of our knowledge, no *Lp2* outbreaks have been reported thus far, only sporadic cases. In Germany in 2008, a case associated with a hotel stay, caused by *Lp2* ST39, has been described [27], while in Italy in 2016, *Lp2* ST1455 was identified in a case with a community-acquired LD (data collected by the Italian national reference laboratory for *Legionella*). By querying the SBT database maintained by Public Health England’s Bioinformatics Unit in November 2020 (Accessed via web archive: https://webarchive.nationalarchives.gov.uk/20190501130700/http://bioinformatics.phe.org.uk/legionella/legionella_sbt/php/sbt_homepage.php; new website under construction at time of publication), we found that ST1455 is poorly represented with only four strains: two from France, one from Switzerland and one from north-eastern Italy, isolated from a community-acquired LD case in 2016 in a region far from the current outbreak area.

In the outbreak described here, we used multiple diagnostic methods combined with a thorough epidemiological investigation to explore all possible common sources of infections, which provided great value in recognising the epidemic event. It is well-documented that no diagnostic test is 100% sensitive and specific and, although the UAT is easy and rapid to perform and highly sensitive for *Lp1* infections, it has limited sensitivity for other *Lp* serogroups [17,20,28-31]. On the contrary, culture of respiratory secretions, which is the only method able to identify single *Lp* serogroups and non-*Lp* species, is seldom performed because of low sensitivity and the need for specific media and experienced microbiologists; further acquisition of respiratory secretions is complicated by the existence of a non-productive cough in LD cases [16]. For these reasons and the higher virulence of *Lp1* compared with non-*Lp1* serogroups, these strains represent only 9% of isolates stored at the national reference laboratory of *Legionella* and, in 2018, amounted to only ca 7.9% of the culture-confirmed LD cases notified in the EU/ European Economic Area (EEA) (personal communication, Lara Payne Hallström, ECDC, September, 2019). In this outbreak, the possibility to discriminate *Lp1* cases from non-*Lp1* cases by real-time PCR combined with the isolatation of *Lp2* colonies by culture suggested that non-*Lp* 1 serogroups were responsible for the epidemic event. Moreover, as already observed in other *Legionella* infections [22], nested-SBT was applied directly to respiratory samples. This method improved the diagnostic identification because it allowed the detection of three additional cases caused by ST1455 strains (six total infections). Therefore, fielding as many diagnostic and typing tools as possible in combination with epidemiological data is of great benefit in outbreak investigations, as demonstarated in this unusual LD outbreak. In particular, the use of real-time PCR has played an important role in identifying the majority of cases of this epidemic event, thereby providing strong justification for performing PCR in pneumonia cases requiring hospitalisation. 

The detection of any species or serogroup of *Legionella* from lower respiratory secretions, lung tissue, pleural fluid or extrapulmonary site by a validated nucleic acid amplification test has been recently adopted by the Centers for Disease Control and Prevention in order to define a confirmed LD case [32]. According to the EU case definition, however, a case with a positive PCR test is only considered a probable LD case and, therefore, laboratories mainly adopt UAT despite the well-known limitations [15]. Therefore, we propose that the case definition of LD should be changed in the EU so that PCR can be used to define a confirmed case and that laboratories are encouraged to use this molecular method. We think this could increase the diagnosis of infection in cases which would otherwise remain underdiagnosed.

The timely collection of respiratory and environmental samples made it possible to find a unique match between the *Lp2* isolates from clinical samples and environmental samples, specifically those from the water collected in the Chiese river and its irrigation canals, typed as ST1455. The natural occurrence of *Legionella* in freshwater systems is well documented [16]. However, with the exception of irrigation canals, we could not identify any process of water uptake from the river that was able to produce aerosol, nor was there any evidence that a source of infection (e.g. industrial drains) had contaminated the river. Of note, an LD outbreak linked to the Wester river and industrial drains was described in 2013 in Germany [33]. On this occasion, the epidemic strain found in water samples from the Wester river and its branches came from two cooling towers of different companies: a sewage pre-treatment plant within the municipal water supply system and the city waste water treatment plants.

According to local informants, irrigation in the area affected by the outbreak mainly occurs through flooding of the cultivated land using water from irrigation canals. However, there was the suspicion that aerosol-producing sprinklers may had been used.

In the period preceding the first LD cases, the river water level was quite low because of high temperatures and low rainfall, meteorological conditions that could have favoured increased *Legionella* concentration in the river. Heavy thunderstorms occurred in the days immediately before the occurrence of the first cases, creating conditions which could have been favourable to the production and spread of infectious aerosols. Similar conditions were observed in an *Lp* outbreak that occurred in the same year between July and August in Lombardy region in the metropolitan area of Milan [34]. Although the area where the cases occurred has many industrial plants equipped with cooling systems, *Lp2* ST1455 colonies were not detected in any of the sampled cooling towers. One explanation could be that news reports about the outbreak were spread through newspaper and social media channels from the beginning. This possibly allowed the owners of industrial plants potentially contaminated by *Legionella* to implement adequate disinfection measures so, by the time analysis was performed, the water samples showed negative results.

During the environmental investigation, our main obstacle in identifying the source(s) of infection was the lack of a register of cooling towers, which, although recommended in Italian guidelines for *Legionella* prevention and control since 2015, has not been adopted by most municipalities.

In the absence of such a register in an affected area, we cannot excluded the possibility that additional, unknown cooling systems also exist and were not inspected. Therefore, the establishment of a cooling tower register was one of the main recommendations made to local and regional health authorities in order to facilitate future outbreak investigations.

In conclusion, in spite of the large amount of clinical and environmental samples analysed and in-depth epidemiological and environmental investigations, the source of infection of this unusual outbreak remains not clearly identified. Notwithstanding, the outbreak led to the establishment of a cooling tower register in the whole region, which marks an important public health policy change.
